# Sodium nitroprusside protects HFD induced gut dysfunction via activating AMPKα/SIRT1 signaling

**DOI:** 10.1186/s12876-021-01934-y

**Published:** 2021-10-02

**Authors:** Xiaomei Li, Chen Li, Yuanqi Li, Cong Liu, Xue Liang, Ting Liu, Zhihua Liu

**Affiliations:** Guangzhou Key Laboratory of Enhanced Recovery after Abdominal Surgery, Innovation Center for Advanced Interdisciplinary Medicine, The Fifth Affiliated Hospital of Guangzhou Medical University, Guangzhou, 510700 China

**Keywords:** AMPKα/SIRT1 signaling, NO, SNP, Intestinal barrier dysfunction, HFD, Gut microbiota dysbiosis

## Abstract

**Background:**

Activation of Adenosine 5′-monophosphate-activated protein kinase/Sirtuin1 (AMPK/SIRT1) exerts an effect in alleviating obesity and gut damage. Sodium nitroprusside (SNP), a nitric oxide (NO) donor, has been reported to activate AMPK. This study was to investigate the effect of SNP on HFD induced gut dysfunction and the mechanism.

**Methods:**

SNP was applied on lipopolysaccharide (LPS) stimulated Caco-2 cell monolayers which mimicked intestinal epithelial barrier dysfunction and HFD-fed mice which were complicated by gut dysfunction. Then AMPKα/SIRT1 pathway and gut barrier indicators were investigated.

**Results:**

SNP rescued the loss of tight junction proteins ZO-1 and occludin, the inhibition of AMPKα/SIRT1 in LPS stimulated Caco-2 cell monolayers, and the effects were not shown when AMPKa1 was knocked-down by siRNA. SNP also alleviated HFD induced obesity and gut dysfunction in mice, as indicated by the decreasing of intestinal permeability, the increasing expression of ZO-1 and occludin, the decreasing levels of pro-inflammatory cytokine IL-6, and the repairing of gut microbiota dysbiosis. These effects were complicated by the increased colonic NO content and the activated AMPKα/SIRT1 signaling.

**Conclusions:**

The results may imply that SNP, as a NO donor, alleviates HFD induced gut dysfunction probably by activating the AMPKα/SIRT1 signaling pathway.

## Background

The gut dysfunction, which is characterized by gut microbiota dysbiosis and intestine epithelium barrier impairment, has a strong link to the development of metabolic disorders such as obesity and type 2 diabetes mellitus [1, 2]. The gut microbiota, which has more than 1200 different bacterial species and 1.5–2.0 kg biomass in the gastrointestinal tract, encodes 500 times more genes than its human host [3]. The gut microbiota plays important roles in ingesta digestion, immunity regulation, and energy equilibrium. The intestine epithelium barrier, which comprises epithelial cells and intercellular tight junction proteins including ZOs, occludins, and claudins, is a multilayered barrier that defends against harmful compounds and pathogens. The tight junction proteins play a vital role in retaining the intestine epithelium barrier. Loss of epithelial tight junction proteins impairs paracellular barrier, resulting in the increasing of the intestine permeability and subsequent intestinal penetration of luminal bacteria and harmful metabolites [4]. Increasing shreds of evidence have shown that gut dysfunction contributes to obesity, and modulation of gut dysfunction is able to improve lipid metabolism and alleviate systemic inflammation in severe obesity [5, 6]. Therefore, amelioration of gut dysfunction is a potential therapeutic measure for obesity and its complicated metabolic syndrome.

Obesity, which is resulted from the high energy intake and low energy expenditure also causes gut dysfunction [5, 7]. AMPK is a crucial kinase that is involved in the regulation of energy metabolism. It has been reported that the activation/phosphorylation of AMPK alleviates obesity and its related metabolic syndrome via activating the downstream effector SIRT1 [8–12]. SIRT1 is a nicotinamide adenine dinucleotide (NAD)-dependent histone deacetylase and its activation by NAD displays an ability to rejuvenate the gut adult stem cells and attenuate the gut damage in aged mice [13]. In addition, AMPK has SIRT1-dependent anti-apoptosis and anti-inflammatory effects [14, 15], and the anti-inflammatory effect of activated/phosphorylated AMPKα is involved in the protection of intestinal barrier [7, 16].

SNP, as a NO donor, is a rapid-acting intravenous vasodilator that has been widely used clinically in hypertensive crises for decades [17–21]. However, recent studies have shown that SNP may also have roles in regulating energy metabolism. SNP increases the phosphorylation of AMPK, specifically AMPKα, then the phosphorylated AMPKα translocates to the nucleus, and then activates gene expression that leads to increasing glucose uptake in the skeletal muscle [22, 23]. NO that is derived from enterocytes has been reported to restore colitis via alleviating tissue damage and macrophage infiltration [24]. However, the role of SNP and NO on obesity-induced gut dysfunction has not been explored.

Due to the crosstalk between gut dysfunction, obesity, AMPK/SIRT1 signaling, and the role of SNP on AMPKα phosphorylation, we hypothesized that SNP protected against HFD induced gut dysfunction via activating AMPKα/SIRT1 signaling. Our experiments on LPS stimulated Caco-2 cell monolayers that mimicked intestine epithelium barrier dysfunction and HFD-fed mice that were complicated by gut dysfunction found that SNP alleviated obesity and gut dysfunction by recovering HFD inhibited AMPKα/SIRT1 signaling.

## Method

### Animals

The male C57BL/6 mice (6w, 20 ± 2 g), normal diet, and high-fat diet feed were provided by the Guangdong Medical Laboratory Animal Center (Foshan, China). Mice were kept under controlled temperature and light conditions (25 °C,12 h light–dark cycle), with free access to food and water. Mice were randomly distributed into three groups containing six animals each: normal diet (ND), high-fat diet (HFD), HFD with sodium nitroprusside (HFD + SNP). Mice were fed with ND or HFD for 16 weeks. SNP (71778, Sigma, USA) was supplemented in drinking water (1 mg/mL) from the fifth week of feeding to the end of the experiment. The study was carried out in compliance with the ARRIVE guidelines 2.0. All methods were performed in accordance with the relevant guidelines and regulations (Directive 2010/63/EU in Europe), and the animal study protocols were approved by committee review of animal experiments in Guangzhou Medical University (Document no. 2018-084).

### Gut microbiota analysis

Gut microbiota analysis was performed as previously described [25]. After 16 weeks of feeding and SNP treatment, feces samples were collected for the 16 S rRNA gene sequencing. Firstly, fecal DNA was extracted using a PowerSoil DNA Isolation kit (12888, Mobio, USA). Then, V3-V4 regions of the 16 S rRNA gene were amplified by PCR with specific primer sets. Next, PCR products were purified and normalized to equal DNA concentration and sequenced using the Illumina Miseq sequencer PE250 (Illumina, USA). The effective reads from all samples were clustered into OTUs based on 99% sequence similarity according to Qiime Uclust. The OTUs were annotated through RDP Classifier (Version 2.2), according to the GreenGene database, then the composition and abundance information of each sample at different classification levels were statistically summarized.

### Intestinal permeability assay

The intestinal permeability assay was performed as previously described [26]. Fluorescein isothiocyanate conjugated dextran (FITC-dextran) (68,059, Sigma, USA) was dissolved in sterile saline (100 mg/mL). Mice were fasted for 12 h and deprived of water for 4 h, then mice were orally gavaged with FITC-dextran (500 mg/kg body weight). 4 h after the gavage, mice were anesthetized with sodium pentobarbital (50 mg/kg body weight), and blood samples were collected from the abdominal aorta. The serum was prepared for fluorescence measurements (excitation, 485 nm; emission, 520 nm) using Thermo Scientific ™ Varioskan ™ LUX (Thermo Scientific, USA). Serum FITC-dextran concentration was quantified against a calibration curve.

### Histology analysis

Mice colon tissues were collected and fixed in 4% formaldehyde, paraffin-embed, sectioned and stained by hematoxylin and eosin (H&E). Histology analysis was performed as previously described [27].

### Cell culture

The human epithelial cell line Caco2 was purchased from American Type Culture Collection (ATCC). Cells were cultured with Dulbecco’s modified eagle medium (DMEM) containing 10% fetal bovine serum in a humidified incubator with 5% CO2 at 37 °C. Caco2 cells were differentiated into polarized epithelial monolayers by culturing on polypropylene membrane on 100 mm culture plates. The Caco2 cells were cultured and passaged successfully before the tests. After passaging for five generations, Caco-2 cells were passaged into 60 mm culture plates at a density of 1 × 10^6^ cells /mL and were subjected to four treatments as follows: Control, lipopolysaccharide(LPS, 50 ng/mL) treatment for 46 h, LPS plus SNP (LPS,50 ng/mL; SNP, 10, 30 ng/mL) for 48 h. After the treatments, cells were harvested for subsequent experiments.

### Western blot analysis

Western blot was performed as previously described [28]. Colon tissues were ground and Caco2 cells were lysed in RIPA buffer, and 50 µg total protein was separated by 12% SDS-PAGE, and then transferred to polyvinylidene fluoride membrane (cas# FFP22, Beyotime Biotechnology, China). The membrane was blocked using 5% skimmed milk powder in PBST (PBS with 0.1% Tween-20) and then was incubated overnight at 4℃ with the following primary antibodies: anti-phospho-AMPKα, anti-AMPK, anti-SIRT1 (cas# 2531s, 8469s·2532s, 1:1000, Cell Signaling, USA), anti-ZO-1, anti-β-actin (cas# ab216880, ab227387, 1:1000, Abcam, USA), and anti-occludin (cas# 66378-1, 1:1000, Proteintech Group, USA). After washed with PBST for 3 times, the membrane was incubated with secondary antibodies (cas# E030120-02, E030110-02, EarthOx, USA) in PBST for 2 h. BeyoECL Moon (cas# P0018FM, Beyotime Biotechnology, China) was used for chemiluminescence and ChemiDicTM XRS + Imaging System was used to take pictures (BioRad Laboratories).

### Transfection of small interfering RNA

The specific and control siRNA sequences were as follows: AMPKα1, sense 5′-CGGGAUCAGUUAGCAACUATT-3′ and antisense 5′-UAGUUGCUAACUGAUCCCGTT-3′; negative control, sense 5′-UUCUCCGAACGUGUCACGUTT-3′ and antisense 5′-ACGUGACACGUUCGGAGAATT-3′ (GenePharma, China). Caco-2 cells were cultured and passaged in the same way as the above procedures, then cells were seed into 60 mm culture plates at a density of 1 × 10^6^ cells/mL. The cells were incubated in Opti-MEM and transfected with each siRNA (100 nM) using Lipofectamine 3000 Transfection Reagent (L3000015, Invitrogen, Carlsbad, CA, USA), according to the manufacturer’s protocol. Transfection of small interfering RNA was performed as previously described [7]. Following the transfection, LPS and SNP were added and co-incubatied in the same way as the above procedures. After the treatments, cells were harvested for subsequent experiments.

### Statistics analysis

Data were subjected to statistical analysis by one-way ANOVA or Student’s T-test using SPSS 16.0. The least significant difference test was selected when the variance was homogeneous, and Dunnett’s T3 test was used when the variance was not homogeneous. Statistical significance was considered when *p < 0.05, **p < 0.01 and ***p < 0.001. All data were expressed as Mean ± *S.E.M*.

## Result

### SNP alleviated LPS-induced intestinal barrier dysfunction via AMPKα/SIRT1 signaling in human Caco2 cells

**Fig. 1:**
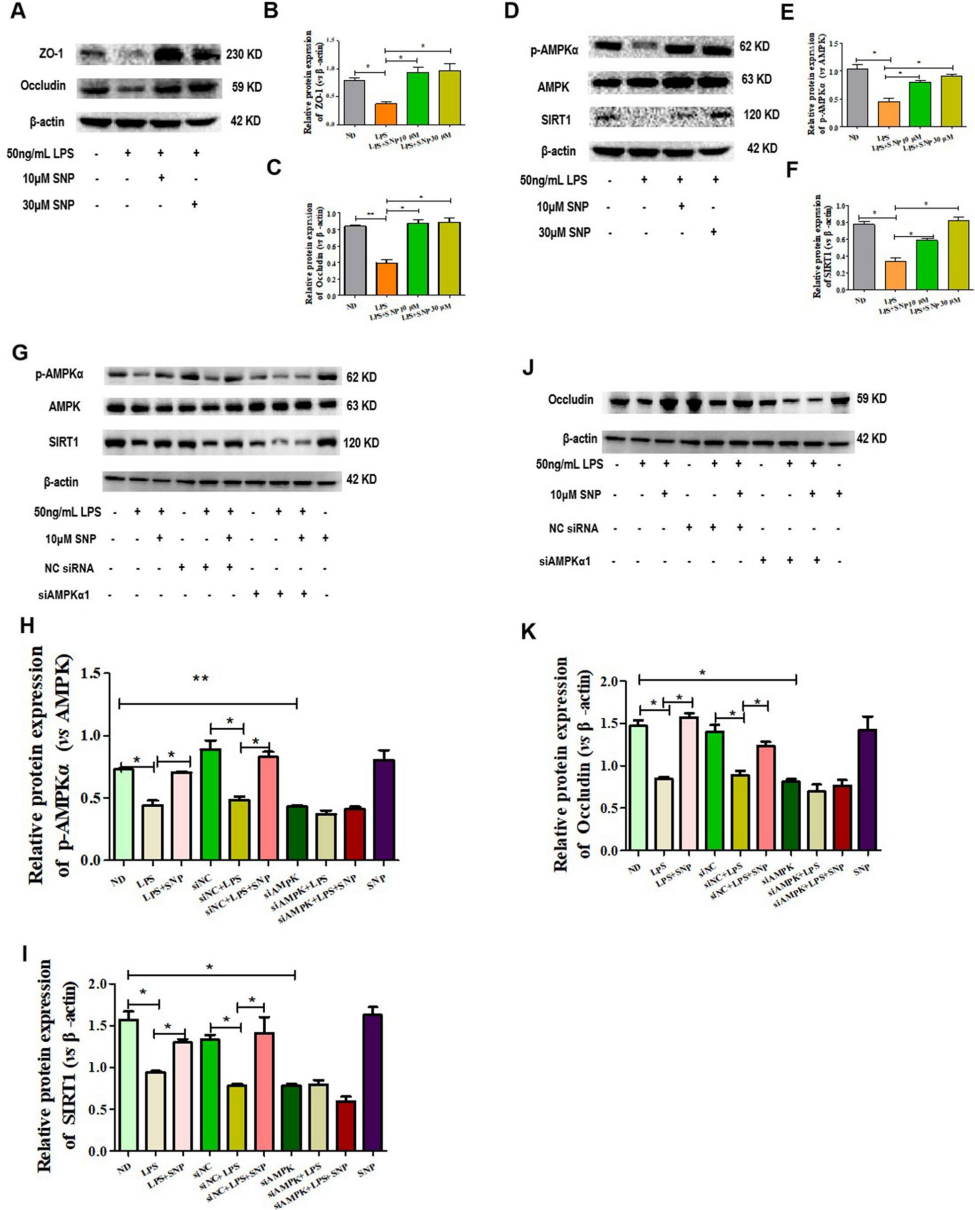
SNP alleviated LPS-induced intestinal barrier dysfunction via AMPKα-SIRT1 signaling. Intestinal barrier dysfunction of Caco-2 cell monolayers was stimulated by 50 ng/mL LPS treatment for 46 h and LPS plus 10 µM/30 µM SNP treatment for 48 h. **A**–**C** Protein levels of intestinal barrier indicators ZO-1 and occludin. (D-F) Protein levels of phosphorylated AMPKα and SIRT1. **G**–**I** Protein levels of SIRT1 and  phosphorylated AMPKα when AMPKα was knocked down. **J**–**K** Protein levels of occludin when AMPKα was knocked down; Data were presented as the mean ± *S.E.M.* (*p < 0.05, **p < 0.01, ***p < 0.001)

Phosphorylation of AMPKα has been reported to be involved in intestinal barrier function [7], and studies have shown that NO plays a role in the activation of AMPK pathway [29–31]. Therefore, we investigated whether SNP, a NO donor, could ameliorate intestinal barrier dysfunction via AMPKα phosphorylation in the human colonic epithelial cell line Caco2. LPS was used to induce intestinal barrier dysfunction in Caco-2 cell monolayers, and as expected, it reduced the expression of tight junction proteins ZO-1 and occludin. 10 µM and 30 µM SNP treatment markedly restored their expression (Fig. 1A–C). LPS also inhibited the phosphorylation of AMPKα in Caco-2 cells, and the inhibition was restored by SNP treatment (Fig. 1D, E). Coincidently, SIRT1, the key player in AMPK mediated anti-inflammatory effects, was also inhibited by LPS and again restored by SNP (Fig. 1D, F). However, when AMPKα was knocked down by siRNA, LPS decreased SIRT1 and occludin could no longer be restored by SNP (Fig. 1G–K). Taken together, these data indicated that SNP alleviated LPS-induced intestinal barrier dysfunction via AMPKα/SIRT1 signaling.

### SNP alleviated obesity of HFD-fed mice

**Fig. 2:**
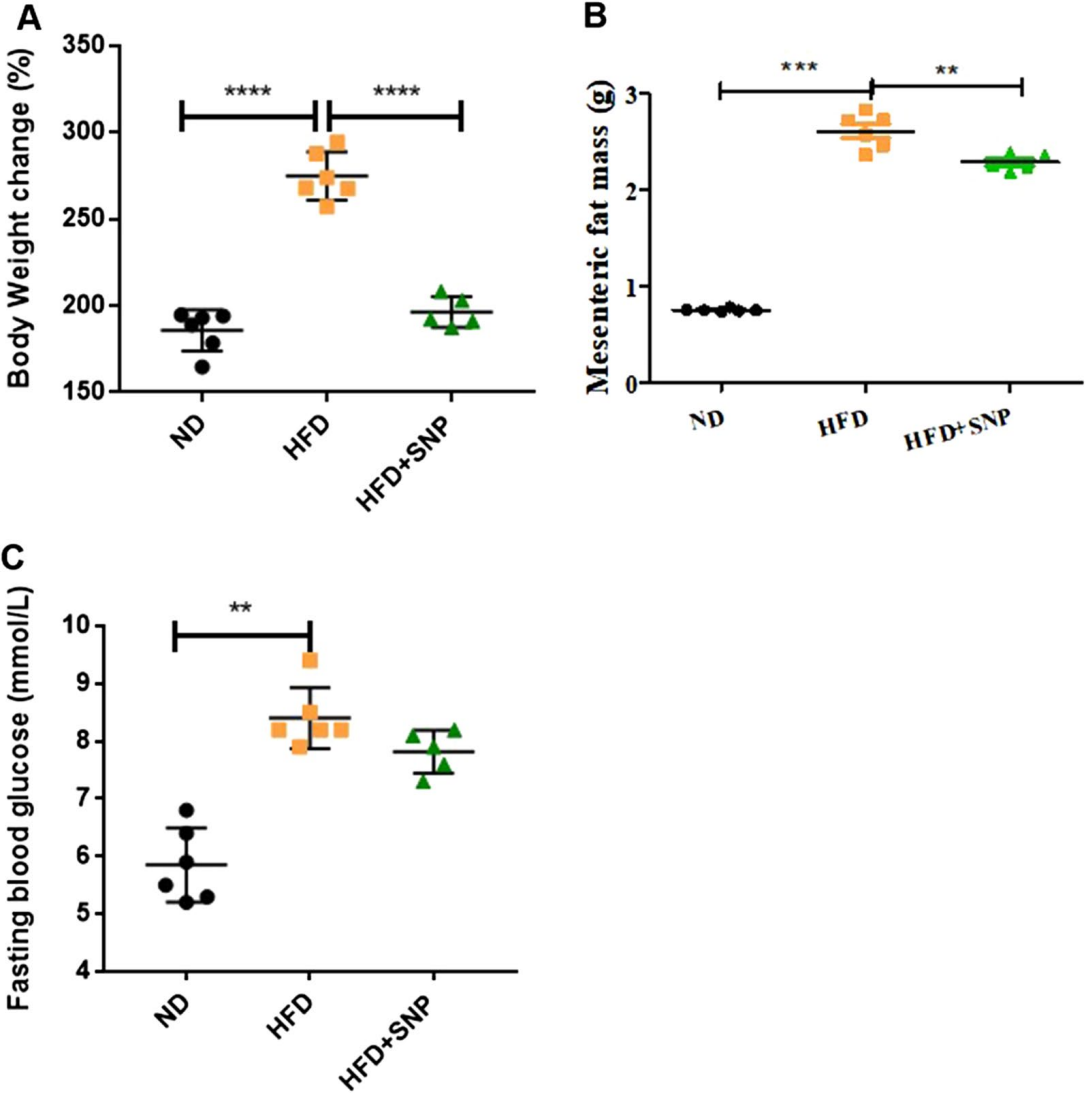
SNP alleviated obesity of HFD-fed mice. Mice (n = 6) were fed with ND or HFD for 16 weeks. SNP was supplemented in drinking water (1 mg/mL) from the fifth week of feeding to the end of the experiment. **A** Body weight changes; **B** Mesenteric fat mass; **C** Fasting blood glucose levels. Data were presented as the mean ± *S.E.M.* (*p < 0.05, **p < 0.01, ***p < 0.001)

The impaired intestinal epithelial barrier has been reported to be associated with the development of metabolic disorders, especially obesity [2, 32–35]. Since SNP alleviated intestinal barrier dysfunction in human Caco2 cells, we then investigated whether SNP could alleviate obesity of HFD-fed mice. As shown in Fig. 2, SNP significantly reduced body weight, mesenteric fat mass and fasting blood glucose in HFD-fed mice (Fig. 2A–C). These results suggested that SNP alleviated obesity and improved metabolic parameters resulted from HFD.

### SNP ameliorated HFD induced gut barrier dysfunction

**Fig. 3:**
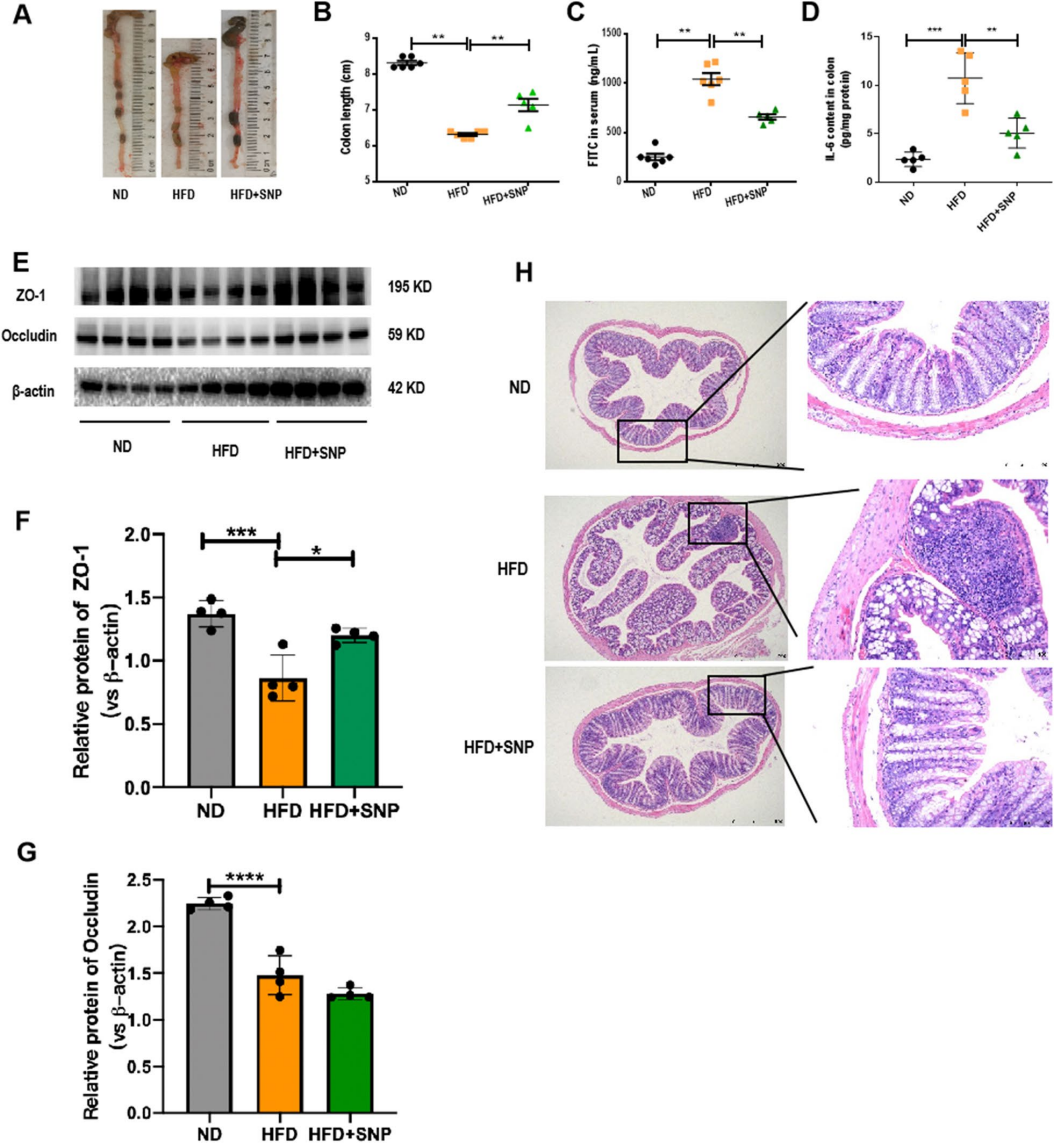
SNP ameliorated HFD-induced intestinal barrier dysfunction. Mice (n = 6) were fed with ND or HFD for 16 weeks. SNP was supplemented in drinking water (1 mg/mL) from the fifth week of feeding to the end of the experiment. **A** Representative pictures of colons of each group; **B** Colon length; **C** Serum FITC-dextran levels; **D** IL-6 levels of colon; **E**-**G** Protein levels of intestinal barrier indicators ZO-1 and occludin (n = 4); (H) Representative histological pictures of hematoxylin and eosin staining. Data were presented as the mean ± *S.E.M.* (*p < 0.05, **p < 0.01, ***p < 0.001)

Following the results that SNP alleviated obesity of HFD-fed mice, we investigated whether SNP could protect gut barrier function of HFD-fed mice. The results showed that colon length was decreased, intestinal permeability was increased, and content of colonic inflammatory factor IL-6 was increased in HFD-fed mice compared with ND-fed mice, while all the damage was ameliorated by SNP (Fig. 3A-D). Moreover, SNP restored the decreased colonic expression of epithelial tight junction protein ZO-1 and occludin of HFD-fed mice (Fig. 3E-G). Consistent with these results, less intestinal mucosal morphological damage and lamina propria leukocyte infiltration were found in SNP-treated HFD-fed mice than those of HFD-fed mice (Fig. 3 H). These results showed that SNP protected against HFD induced intestinal barrier dysfunction and intestinal inflammation.

### SNP resisted HFD inhibited AMPKα/SIRT1 signaling in mice colon

**Fig. 4:**
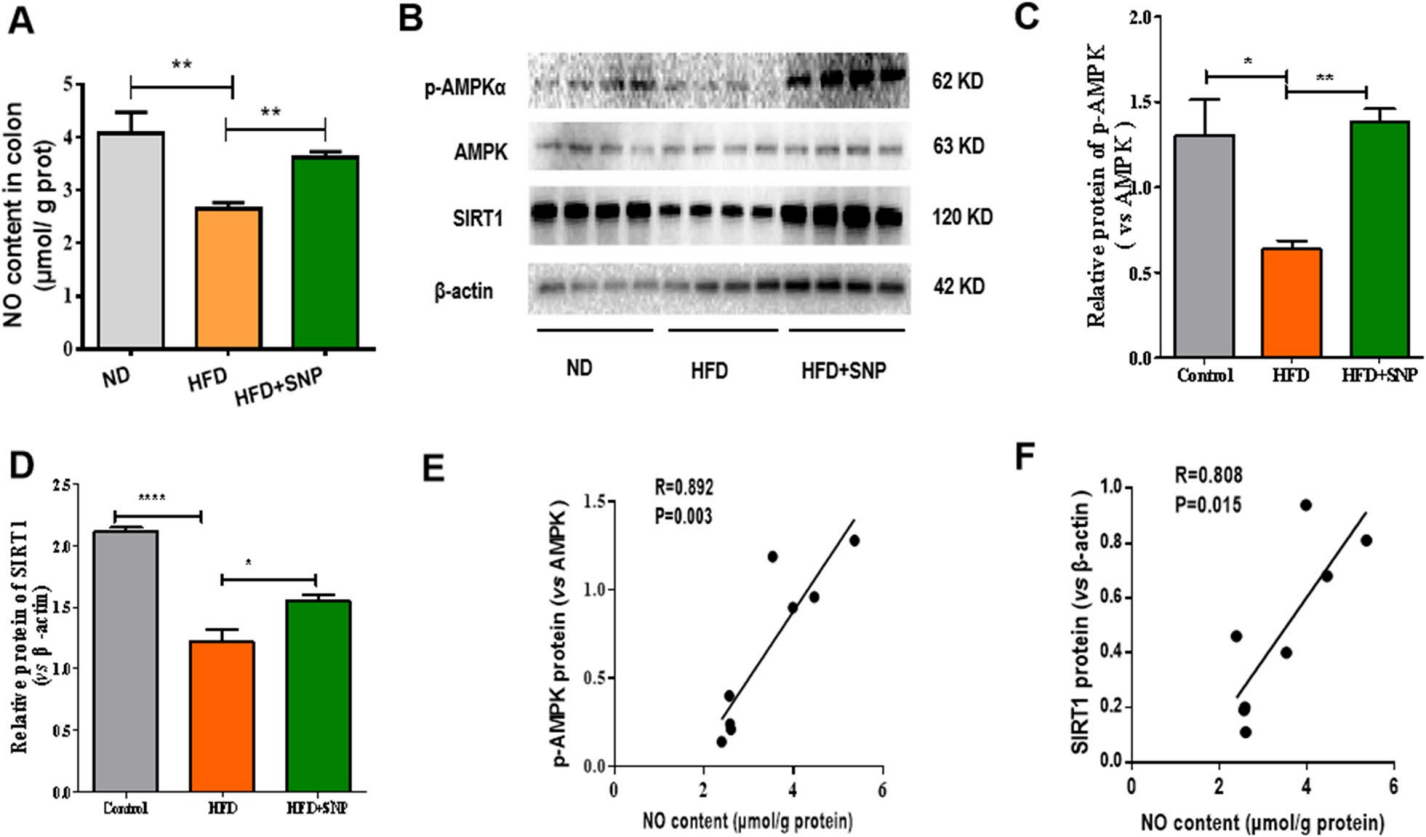
SNP resisted HFD inhibited colonic AMPKα/SIRT1 signaling. Mice (n = 6) were fed with ND or HFD for 16 weeks. SNP was supplemented in drinking water (1 mg/mL) from the fifth week of feeding to the end of the experiment. **A** NO levels of colon; **B**–**D** Protein levels of phosphorylated AMPKα and SIRT1 (n = 4); **E** Correlation between colonic NO content and phosphorylated AMPKα; **F** Correlation between colonic NO content and SIRT1. Data were presented as the mean ± *S.E.M.* (* *p* < 0.05, **p < 0.01, ***p < 0.001)

To verify whether the anti-obesity and intestinal barrier protection effects of SNP on HFD-fed mice were also mediated by AMPKα/SIRT1 signaling, we tested the colonic expression of these signals. Colonic NO level was reduced in HFD-fed mice compared with ND-fed mice, and SNP treatment increased the colonic NO level of HFD-fed mice (Fig. 4A). Protein levels of SIRT1 and phosphorylated AMPKα were lower in the colon of HFD-fed mice, but these alterations were restored by SNP (Fig. 4B-D). Linear regression analyses showed a significant positive correlation of the NO content and the protein levels of SIRT1 and phosphorylated AMPKα (Fig. 4E-F). These results indicated that SNP rescued colonic AMPKα-SIRT1 signaling suppressed by HFD.

### SNP ameliorated HFD induced gut dysbiosis

**Fig. 5:**
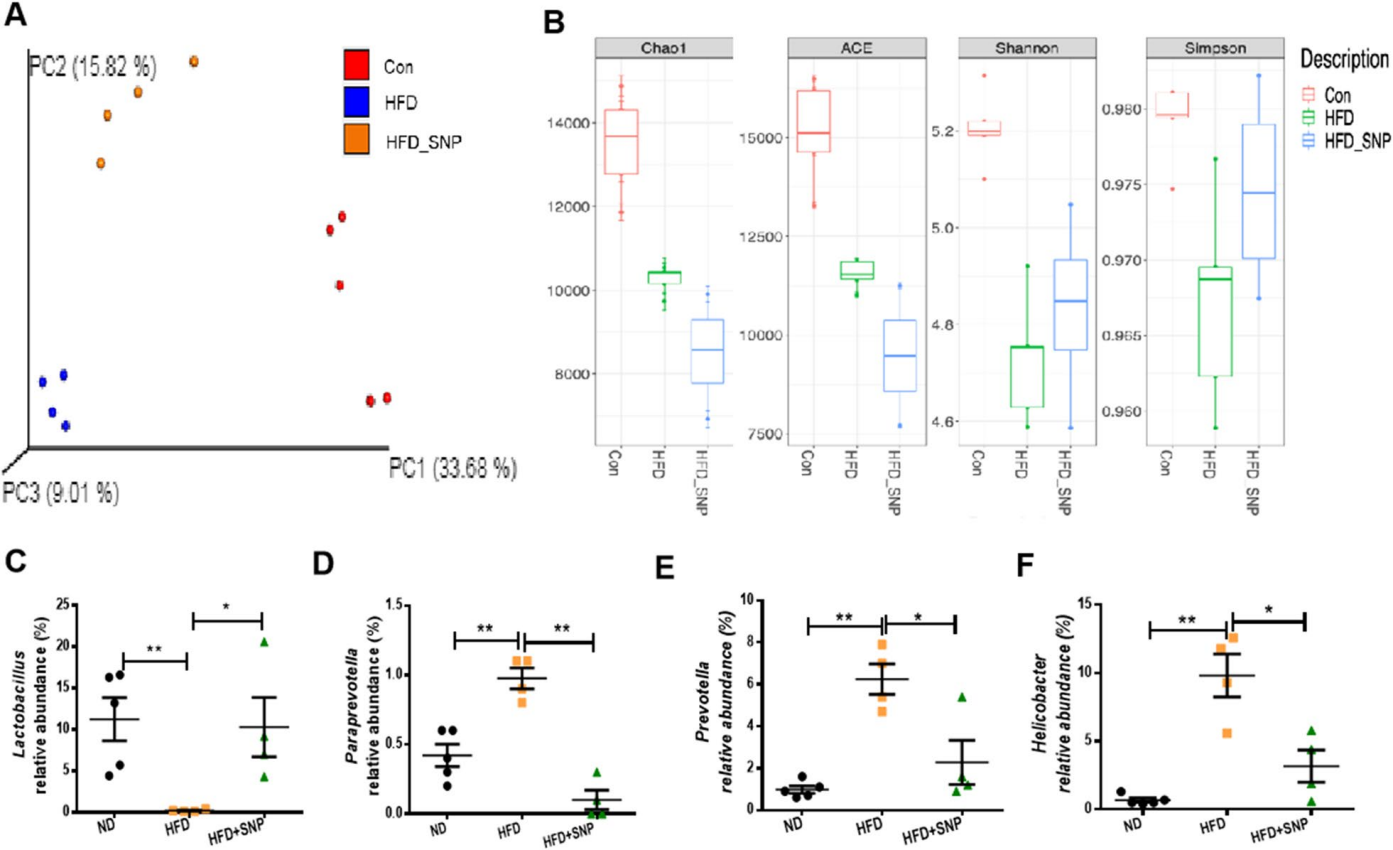
SNP alleviated HFD-induced gut microbiota dysbiosis. Mice (n = 6) were fed with ND or HFD for 16 weeks. SNP was supplemented in drinking water (1 mg/mL) from the fifth week of feeding to the end of the experiment. **A** Principal coordinate analysis (PCoA) plots of fecal microbiota; **B** Richness (Chao1 and ACE indexes) and diversity (Shannon and Simpson indexes) of microbial communities; **C**–**F** Relative abundance of *Lactobacillus, Paraprevotella*, *Prevotella*, and *Helicobacter*. Data were presented as the mean ± *S.E.M.* (* *p* < 0.05, **p < 0.01, ***p < 0.001)

Previous studies have shown that obesity and gut barrier dysfunction is associated with gut microbiota dysbiosis [36, 37]. We next investigated whether SNP had effects on gut microbiota dysbiosis of HFD-fed mice. As shown in Fig. 5A, HFD caused significant changes of gut miacrobiota profiles compared with ND while SNP treatment modulated the profiles of gut microbiota. HFD significantly decreased microbiota richness and diversity, SNP reversed the changes of the diversity but not richness (Fig. 5B). At the genus level, HFD significantly reduced the relative abundance of *Lactobacillus* and increased the relative abundance of *Paraprevotella*, *Prevotella*, and *Helicobacter*, whereas SNP reversed the changes of these microbes (Fig. 5 C-F). So, SNP rescued part of HFD induced gut microbiota dysbiosis.

## Discussion

NO is not only a key regulator of vascular endothelial homeostasis, but may also play roles in intestinal epithelial function. Previous studies have demonstrated that enhancing the bioavailability of NO in enterocytes attenuates colitis by meliorating colorectal inflammation and gut microbiota dysbiosis [38]. A recent study reported that endogenous NO derived from enterocytes ameliorated colitis by alleviating inflammation, macrophage infiltration, and tissue damage. Moreover, induction of NO production in enterocytes with NO precursors inhibited colon cancer by alleviating epithelial barrier damage and inflammation [39]. Our results showed that NO content was significantly decreased by HFD compared with ND, whereas SNP supplementation increased NO content in colon tissue of HFD-fed mice, and then confered protective effects on HFD induced gut dysfunction. The role of SNP in alleviating intestinal barrier damage was also reported in ischemia/reperfusion injury mice models [40–42].

Tight junction proteins are key components of the epithelial barrier and loss of them leads to elevated intestinal permeability and inflammation [43]. Loss of tight junction proteins was found in endotoxin LPS stimulated enterocyte [44–49]. HFD, which produces LPS also leads to loss of tight junction proteins. Our results showed that SNP not only restored LPS-induced loss of the tight junction proteins ZO-1 and occludin in Caco-2 cell monolayers, but also restored HFD induced loss of the tight junction proteins ZO-1 and Occludin in mice, which reversed the increased intestinal barrier permeability.

The impaired intestinal epithelum barrier has been considered to be a pathophysiological factor of metabolic diseases, such as obesity [50–52]. Gut microbiota dysbiosis, which promotes intestinal barrier disruption, contributes to the development of metabolic disease [53–55]. Meanwhile, the metabolic disease also causes gut dysfunction and then leads to gut problems such as colitis. So, gut dysfunction and obesity may interact as both cause and effect. Anyhow, NO is a pleiotropic signaling molecule that involves in regulation of both the intestinal barrier function and the metabolic deseases.[56, 57]. Consistently, we found that NO donor SNP protects against gut dysfunction, as well as obesity.

AMPK signaling has been reported to be involved in endothelial homeostasis [58], Inhibition of AMPK promotes epithelial barrier dysfunction [7], while the activated/phosphorylated AMPKα protects against colitis via alleviating intestinal barrier dysfunction and anti-inflammation [7, 16]. The effect of prebiotics, fructo-oligosaccharides (FOS) for example, in protecting intestinal epithelial tight junction has been reported to be mediated by AMPK activation[59]. The role of NO in endothelial homeostasis has been reported to be mediated by AMPK signaling. Increased NO derived from low-concentration SNP increases levels of phosphorylated AMPK and its activity in endothelial cells [60]. L-arginine, another precursor for NO synthesis, has been reported to increase the expression of tight junction proteins and improve intestinal barrier function by activating of AMPK signaling in rat small intestines and IEC-6 cells during heat stress [61]. Consistent with the previous reports, we found that NO donor SNP restored LPS and HFD induced loss of tight junction proteins via AMPKα-SIRT1 signaling in Caco-2 cell monolayers and HFD-fed mice. What’s more, NO content and the activated AMPKα/SIRT1 signaling were positively correlated in mice’s colons.

The present study didn’t include a SNP control on ND-fed mice. So if SNP reduced the basal mesenteric fat mass and blood glucose could not been shown here. Effects of SNP or other NO-generating compounds in regulating blood glucose and insulin sensitivity have been tested sufficiently on cell and animal models of diabetes and human with diabetes, and all the results have shown that treatment of NO donor SNP or NO precursor L-Arginine reduce blood glucose levels, improve insulin resistance and lipid profiles in diabetic models [62–68], however, they do not affect blood glucose, lipid profile in non-diabetic group [69, 70]. So, we inferred that SNP might act specifically on subjects with metabolic problems while have little effects on normal subjects, however, this assumption need further investigation.

In conclusion, our study indicated that SNP, as a NO donor, which has been applied as an anti-hypertensive drug, had protective effects on epithelial barrier dysfunction and gut microbiota dysbiosis resulted from HFD. It was noted that SNP alleviated HFD induced intestinal epithelium barrier dysfunction and inflammation by activating the AMPKα/SIRT1 signal, and these effects were dependent on the increased NO content derived from SNP. These results implied that SNP might be a potential drug for patients with gut dysfunction.

## Data Availability

Sequence data of the raw paired-end reads were deposited to NCBI/Sequence Read Archive and are accessible through the accession number PRJNA674745, or http://www.ncbi.nlm.nih.gov/bioproject/674745.

## Abbreviations

AMPK: Adenosine 5′-monophosphate-activated protein kinase
SIRT1: Sirtuin1
SNP: Sodium nitroprussiate
NO: Nitric oxide
IV: Intravenous
HFD: High-fat diet
LPS: Lipopolysaccharide
ZO-1: Zonula occludens 1
siRNA: Small interfering Ribonucleic Acid
IL-6: Interleukin- 6
NAD^+^: nicotinamide adenine dinucleotide
ND: Normal diet
PCR: Polymerase chain reaction
OTUs: Operational taxonomic units
RDP: Ribosomal Database Project
FITC-dextran: Fluorescein isothiocyanate conjugated dextran
H&E: Hematoxylin and eosin
siNC: Negative control small interfering RNA
siAMPKα1: AMPKα1 small interfering RNA
